# Review of collagen type I-based hydrogels: focus on composition-structure-properties relationships

**DOI:** 10.1038/s44385-025-00018-w

**Published:** 2025-05-03

**Authors:** Mariana Pires Figueiredo, Silvia Rodríguez-Fernández, Francesco Copes, Diego Mantovani

**Affiliations:** Laboratory for Biomaterials and Bioengineering, Canada Research Chair Tier I for the Innovation in Surgery, Department of Min-Met-Materials Engineering and Regenerative Medicine, CHU de Quebec Research Center, Laval University, Quebec City, QC Canada

**Keywords:** Natural products, Proteins

## Abstract

This review explores each stage of Collagen type I (Coll-I) hydrogel development, highlighting how sourcing, extraction, solubilization, and modification (e.g*.*, blending, crosslinking, and composite formation) influence its gelation, and structural, mechanical, and biological properties. By clarifying key interrelations among these characteristics, this work serves as a valuable guide for scientists designing next-generation of Coll-I-based hydrogels with optimized properties for tissue engineering and regenerative medicine applications.

## Introduction

Collagen type I (Coll-I) has been widely studied and commercialized as hydrogels for cosmetic and medical applications^1^. This is due to its status as the most abundant extracellular matrix (ECM) protein in animals^2^. In humans, 90% of collagen comprises type I, which is found in both hard (e.g*.*, bones) and soft (e.g*.*, skin) tissues^3^. Coll-I might be extracted from different animal sources, such as mammals or fish. As a conserved protein^4^, Coll-I from other animals closely resembles its human counterpart, making it inherently compatible with human tissues and systems^5^.

In cosmetics, Coll-I is used as a component in foams and creams for skin due to its moisturizing and film-forming properties^6^. In clinics, commercial Coll-I-based products include homeostatic hydrogel or sponge wound dressings, and guided tissue regeneration membranes^7–9^. Moreover, there is an increased interest in expanding collagen use to tissue engineering and regenerative medicine (TERM) for the development of hydrogels to treat damaged tissue due to accidents or diseases, such as fractured bones, damaged cartilage or cardiac tissues, and burned skin. Compared to other Coll-I forms, hydrogels mimic closely the ECM environment and possess the advantage of allowing less invasive administration routes, since the Coll-I viscous solution, called pre-gel, can even be injected and solidify in situ.

For TERM applications, the following are the specific requirements that Coll-I hydrogel must fulfill^10^:

1. *Processability*: Coll-I pre-gel must the able to be assembled into a hydrogel and provide an appropriate shape when injected or implanted in the body.
2. *Biological performance*: Coll-I hydrogels must provide a suitable environment where cells can be embedded or migrate inside after hydrogel implantation and support cell growth. Cell embedding can accelerate the process of scaffold cell colonization and the consequent scaffold remodeling and tissue formation during the tissue healing process.
3. *Mechanical properties*: Coll-I hydrogels must have a similar response to load-bearing and mechanical stress compared to the native and healthy tissue for proper new tissue formation, temporally supporting tissue physically as well as biological functions, such as cell attachment and growth capacity.

Although Coll-I hydrogels are extensively investigated for TERM, their poor mechanical properties limit their use^11^. Therefore, crosslinking, blending with other polymers, or combining Coll-I to particles forming composites, have been widely reported in an attempt to enhance their mechanical properties to match those of the surrounding tissue in the implant^12^. For that, the type and quantity of reactive lateral groups of amino acids (AA) that compose the extracted Coll-I play a pivotal role in understanding and tuning chemical and physical interactions related to hydrogel processing and performance. Nevertheless, for the development of Coll-I hydrogels, the literature usually poorly addresses the source and/or methodology employed to obtain Coll-I, rather focusing on mechanical and biological validation and missing the compositional-structural characterization of the initial raw material. In addition, Coll-I composition is also directly related to physical-chemical parameters that are key in processing Coll-I into hydrogels. This can, ultimately, influence mechanical and biological properties. For instance, some works focusing on Coll-I extraction and characterization have reported a link between the composition of the extracted Coll-I (extColl-I) with the animal age^13^, the animal type^14^, the extraction methods^15^, and the related physical-chemical properties, in particular the isoelectric point (IP) and solubility of the protein in an aqueous medium.

However, the connection between Coll-I sourcing, extraction, and isolation, and their influence on extColl-I physical-chemical properties and processing, remain largely overlooked. Therefore, the main goal of this work is to contribute to filling these gaps and to understand the parameters involved in all the steps of the development of Coll-I hydrogels, from collagen extraction to hydrogel tunning, as well as their interrelations. This knowledge can help to achieve a polyvalent platform for designing materials with tailored properties for specific applications in TERM.

**Fig. 1:**
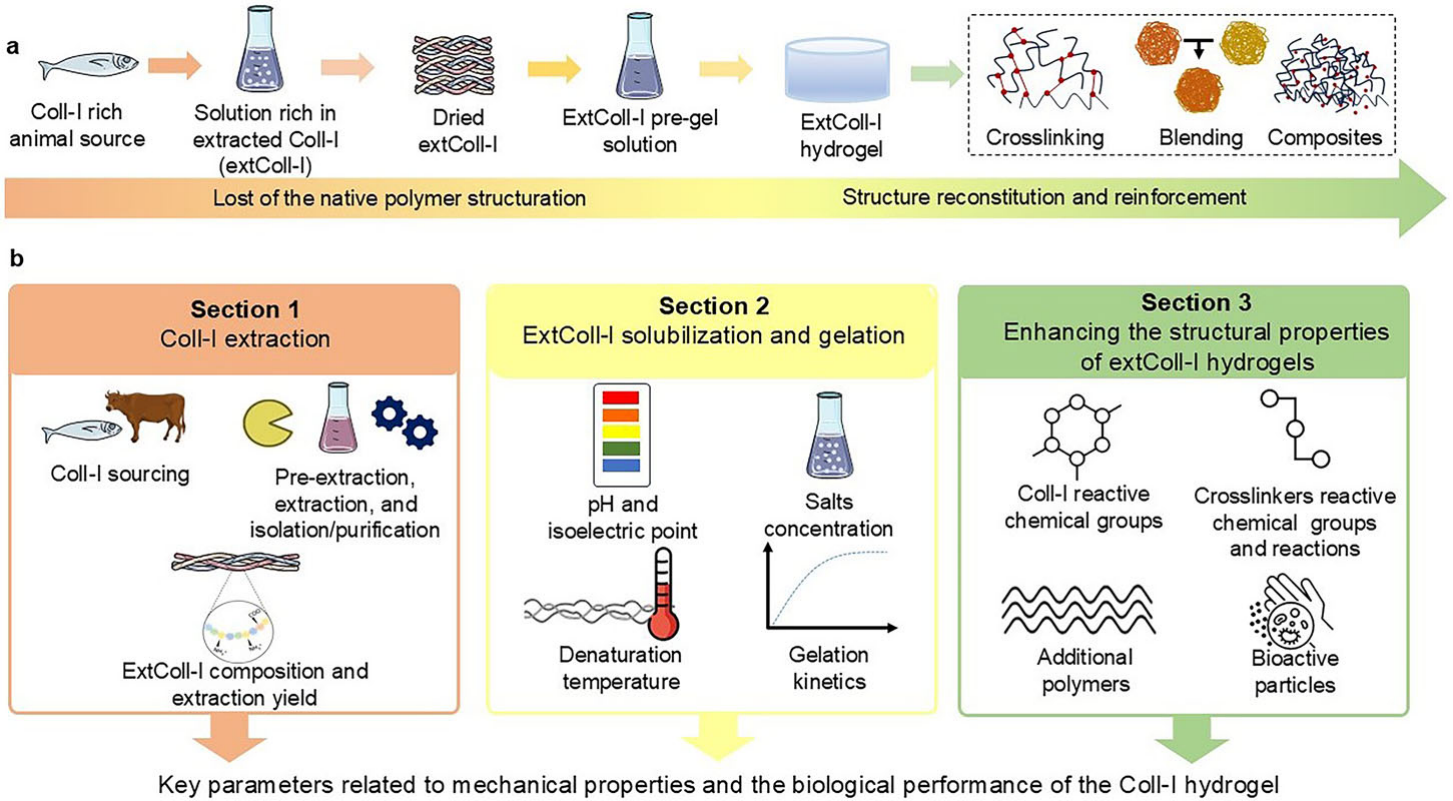
Engineering Coll-I hydrogels. Step by step (**a**). Paper organization and content (**b**)—SECTION 1: Coll-I extraction methods, SECTION 2: Coll-I solubilization and gelation parameters (2), and SECTION 3: Structural modifications for structuring Coll-I hydrogels. Key parameters and aspects presented in sections 1-3 are interconnected and influence the mechanical performance and the biological properties of Coll-I hydrogels. This Figure was partly generated using Servier Medical Art, licensed under Creative Commons Attribution 4.0.

This review is divided into three sections (Fig. 1). *Section 1* presents the animal’s sources and details the most employed tissues for Coll-I extraction, as well as the physical, chemical, and enzymatic processes employed in Coll-I pre-extraction, extraction, and isolation. *Section 2* focuses on variables affecting solubilization and gelation (i.e., animal source, animal tissues, extraction method, pH and IP, salt concentration, and heating) of the extColl-I. In *Section 3*, three techniques (crosslinking, blending, and composites) commonly used to enhance structural properties of extColl-I hydrogels are detailed and discussed. Finally, it is shown that the parameters involved in each step of Coll-I hydrogel development affect their physical-chemical, mechanical, and biological properties.

## Methodology

**Fig. 2:**
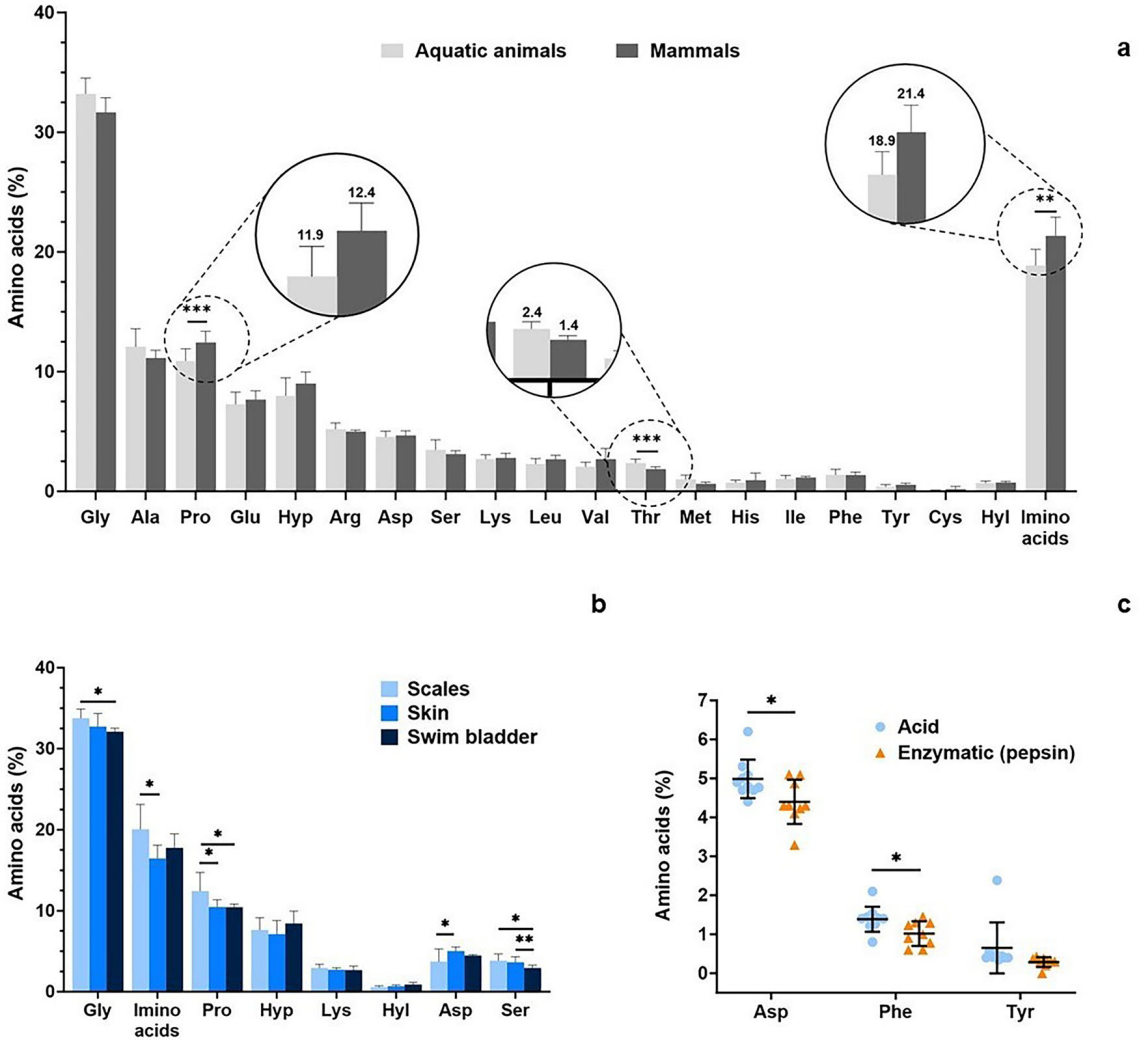
Amino acid composition of the extracted Coll-I. Average amino acid composition of extracted Coll-I based on (**a**) animal type (aquatic non-mammal animals and mammals), and (**b**) on fish parts of the body (scales, skin, and swim bladder); Tyr, Asp, and Phe amino acid content for the skin of aquatic non-mammal animals according to the extraction method (**c**)^26,27,34,84,88,90,93,95,99,100,102,104,111,117,120,121,124,126,128,134,137,159^. *: *p* < 0.1, **: *p* < 0.05, and ***: *p* < 0.01. Data was collected from the cited articles in this caption.

**Fig. 3:**
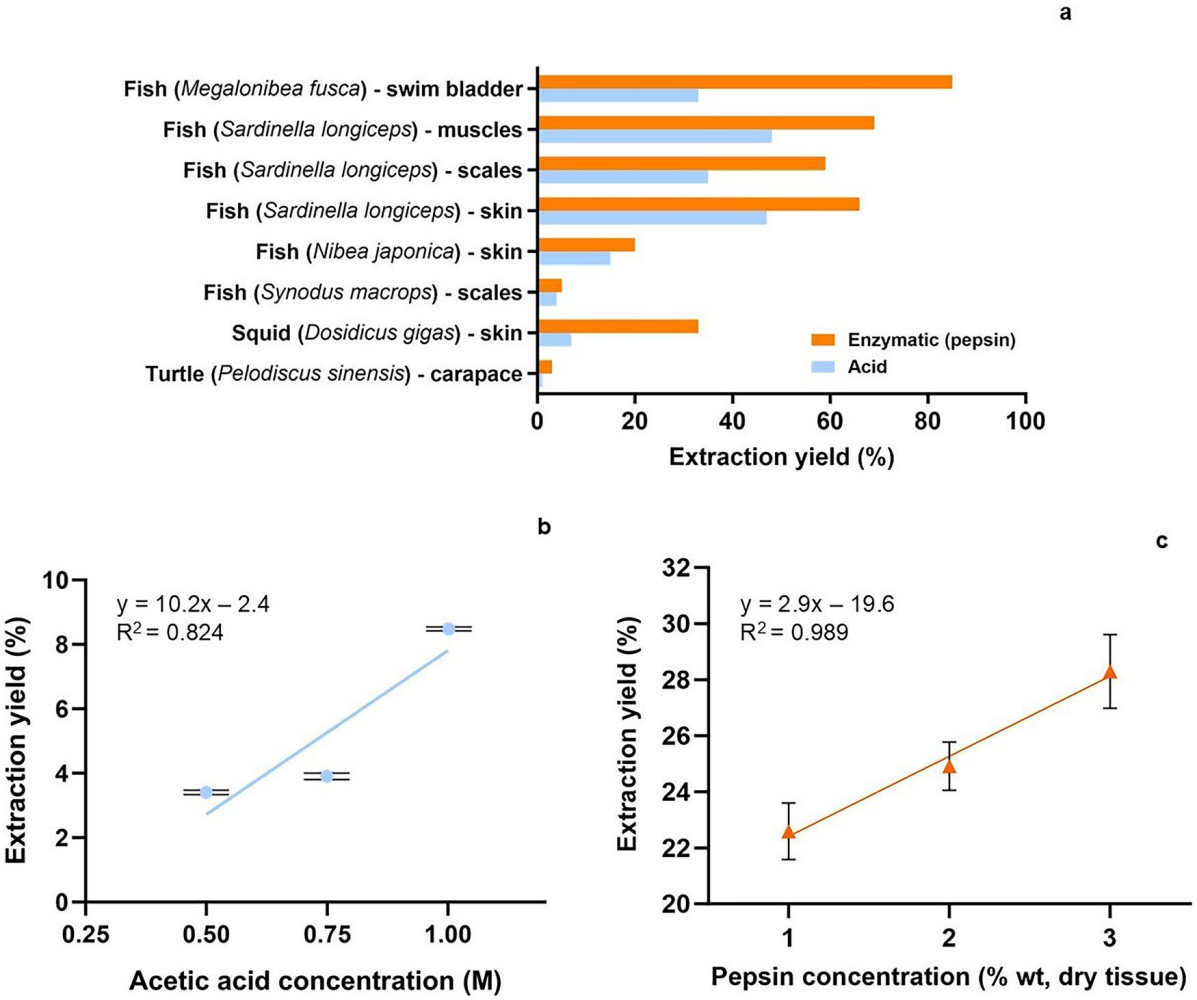
Extraction yields of Coll-I depending on animal type, part of the body, and extraction method. Extraction yields (ratio between the dry weight Coll-I and the dry weight of the tissue) according to (**a**) the extraction method^34,83,109,128,129,159^, acid concentration^108^ (**b**), and pepsin concentration^108^ (**c**). Data was collected from the cited articles in this caption.

**Fig. 4:**
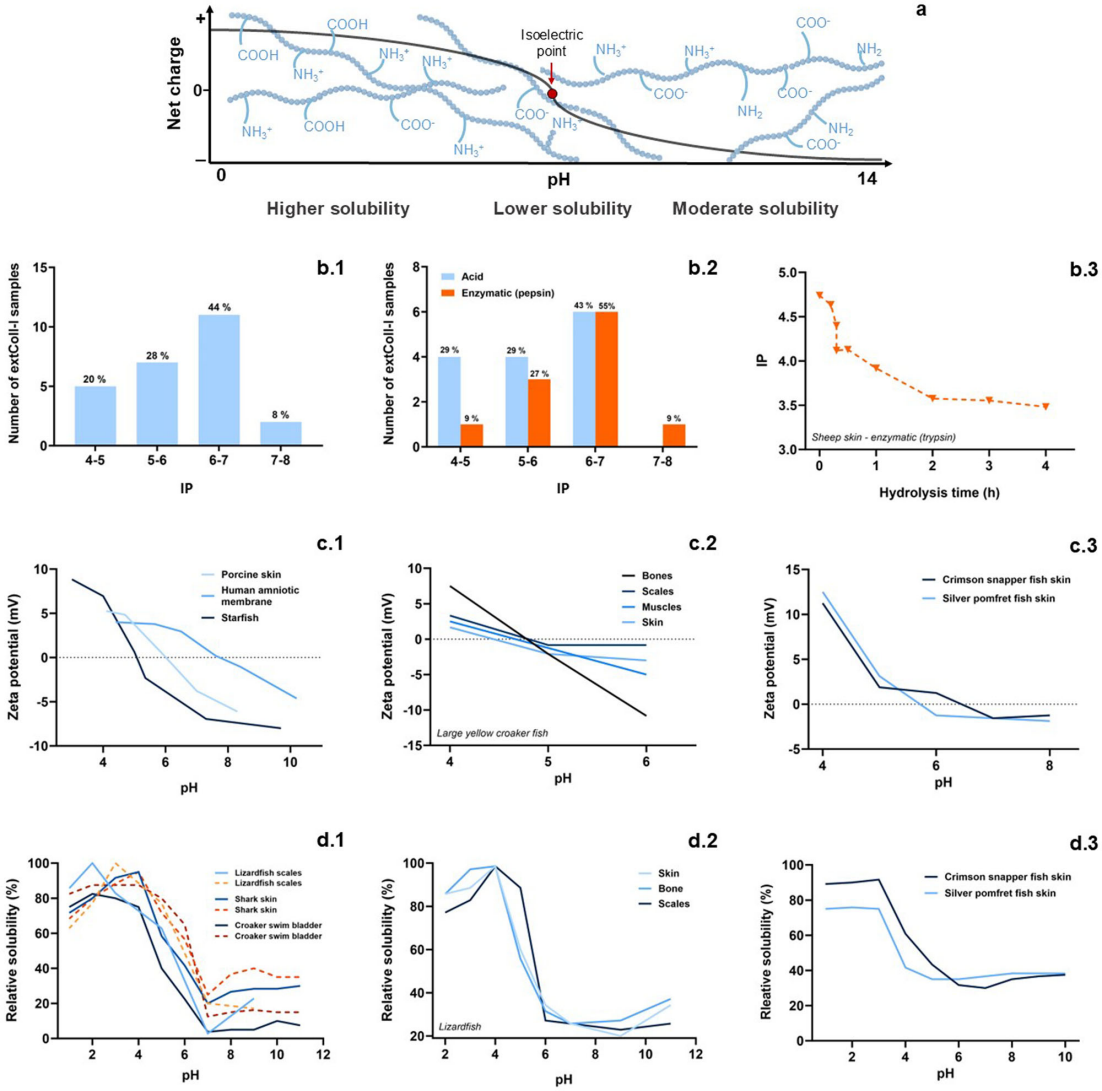
Effect of the pH on the solubilization of extracted Coll-I. Solubility of Coll-I in aqueous medium according to the pH of the medium, related to the residual charge (positive at lower pH values, zero at the isoelectric point, and negative at higher pH values) from amino acids containing chargeable amine (-NH_2_/-NH_3_^+^) or carboxylic (-COOH/-COO^-^) lateral groups (**a**). Overall IP variation (**b.1**) and overall IP variation discriminated by extraction method (**b.2**)^26,28,31,34,37,92,99,104,107,117,128^. Example of the effect of collagen enzymatic (trypsin) hydrolysis on IP of Coll-I^160^ (**b.3**). Examples of IP (equal to the pH in which the zeta potential is zero) variation with the animal type^161^ (**c.1**), tissue type^37^ (**c.2**), and animal species^92^ (**c.3**). Examples of solubility of Coll-I according to the pH for collagen extracted by different methods^35,99,125^ (**d.1**), from different tissues^14^ (**d.2**), and from the same type of animal but different species^92^ (**d.3**). Blue and orange curves refer to acid and enzymatic extraction or treatment of collagen type I, respectively. **b1**, **b2**, **b.3**–**d** present data collected and reproduced from data shown in the references cited in this caption, respectively.

Web of Science (as the wider scientific database) and PubMed (as a biomedical-focused database) were selected to identify scientific papers, through keyword criteria, published between January 2018 to December 2023. The following keywords and Boolean query were used: “(collagen type I OR type I collagen) AND (extraction OR isolation) AND (gel* OR hydrogel*). Only full papers were selected for analysis; review articles, book chapters, proceeding papers, and other types of productions were excluded. After removing duplicates, a screening considering title, abstract, and full text was performed to exclude articles on collagens other than Coll-I, commercial Coll-I, and collagen-derived materials, such as gelatine. Thus, 75 articles were identified. For each article, pertinent information about the animal source (body site, organs, or tissue), and extraction procedure (i.e*.*, the pre-extraction, extraction, isolation methods, and extraction yield) was collected. When available, amino acid content, and physical-chemical parameters of the extColl-I (i.e*.*, IP, solubility as a function of the pH and ionic strength of the solution, and denaturation temperature) were also collected. Data from these papers are displayed in Tables 1 and 2, and Figs. 2, 3, 4b.1–b.3, c.2, c.3, d.1–d.3, and 6. Among the 75 papers, no paper was found covering both the effect of Coll-I composition and physical-chemical properties on the processing and mechanical and biological validation of Coll-I hydrogels. Thus, additional searches were performed with various keywords (not specified here) to assess the influence of the cited parameters on gelation, the effect of animal type, tissue, extraction method, and structural modifications (i.e*.*, crosslinking, blending, composites) on mechanical and biological properties.

Data on AA content in Coll-I from papers resulting from the systematic search were treated. A statistical analysis of the content of AA was conducted to detect possible differences between sources and extraction methods using GraphPad 10.5. For AA comparison between Coll-I extracted from aquatic animals and mammals, the non-parametric Mann-Whitney test (with *n* values equal to 6 and 37 for mammals and aquatic animals, respectively) was used after confirming that the groups did not follow a normal distribution. For AA comparison between Coll-I extracted from different tissues of non-mammal aquatic animals using an acid extraction method, the non-parametric Kruskal-Wallis test, followed by the post hoc Dunn test, was used to compare each AA individually between groups. The *n* values were equal to 4, 6, and 9 for scales, swim bladder, and skin, respectively. To compare the content of the three AA related to pepsin cleavage (Asp, Phe, and Tyr) in Coll-I extracted from the skin of aquatic animals using pepsin (*n* equal to 9) and acid (*n* equal to 10), the non-parametric Mann-Whitney test was applied after confirming that not all groups met the parametric assumptions. Given the high data variation, which necessitates working with larger number of samples, exploratory statistics were performed to identify general trends. Thus, a *p* value of 0.1 or lower was considered significant.

## SECTION 1: Coll-I extraction

### The main sources of Coll-I

In recent literature, aquatic non-mammal animals, represented mainly by fish, were found to be the most studied source of Coll-I (76%), followed by mammals (19%), as shown in Table 1, and skin the most studied tissue source of Coll-I from both aquatic and mammals. Fishes’ higher abundance in terms of global consumption and higher percentage of waste are cited aspects that can justify greater occurrence^16^.

**Table 1:** Overview of Coll-I sources and extraction methodology commonly used

Animal sources		
Aquatic animals (non mammals)	76%	
Mammals	19%	
Birds	3%	
Reptiles	1%	
Amphibia	1%	

Parts of the body	Aquatic animals	Mammals
Scales	19%	—
Swim bladders	7%	—
Fins	1%	—
Muscle	6%	—
Skin and bone	1%	—
Skin	60%	57%
Bone	6%	11%
Tendon	—	27%
Muscular fascia	—	5%
Ears	—	5%
Feet	—	5%
Meat	—	5%
Slaughter by products	—	5%

Extraction method	Aquatic animals	Mammals
Acid	57%	27%
Pepsin	36%	61%
Heating	2%	4%
Pepsin + Ultrasound	1%	4%
Acid-Pepsin	2%	—
Acid-Pepsin-Acid	1%	—
Base	1%	—
Microwave irradiation	—	4%

Sources of collagen type I (Coll-I), and parts of the body or tissues and methodology used to extract it from non-mammal aquatic animals and mammals^13,14,76,79–139^. Data was collected from the cited articles in this caption.

### Steps involved in the extraction of Coll-I and their specificities according to animal and tissue type

The procedure for extracting Coll-I involves three main steps: Pre-extraction, extraction, and isolation/purification. These processes require the action of different reagents and/or enzymes, complemented by physical methods, as detailed in Table 2. Pre-extraction consists of separating non-collagen proteins from Coll-I rich matrix using a basic solution, usually NaOH solution, decalcifying mineralized tissues, such as bones and scales, with acid solutions, as well as de-fatting and de-pigmenting tissues like skin. Thus, tissue type highly influences the pre-extraction treatment choice. It is important to be careful with the use of NaOH alkaline solution since it can also hydrolyze collagen along with other non-collagen proteins. Collagen hydrolysis is expected to increase with the increase in NaOH solution concentration and with the increase of the pre-extraction temperature^17^. After the pre-extraction treatment, Coll-I becomes easy to extract from the tissues.

**Table 2:** Chemical species used to obtain Coll-I

General chemical classification	Chemical agent or physical treatment	Function	Ref.
*Pre-extraction treatments*			
Inorganic base	Sodium hydroxide (NaOH)	Defatting and removal of non-collagen components	^109^
	Sodium bicarbonate (NaHCO_3_)		^85^
Inorganic acid	Hydrogen chloride (HCl)	Demineralization (decalcification)	^83,140^
	Sulfuric acid (H_2_SO_4_)		
	Phosphoric acid (H_3_PO_4_)		
Chelant	Ethylenediaminetetraacetic acid (EDTA)		^124^
Inorganic peroxide	Hydrogen peroxide	Depigmentation	^141^
Organic solvents	Butyl alcohol	Defatting	^83,106,127^
	Butanol		
	Isopropanol		
	Methanol	Defatting and removal of saccharides	^83^
	Ethanol		^90^
*Extraction*			
Organic acid	Acetic acid	Collagen extraction through hydrolysis of polypeptide bonds	^106,109,128^
	Lactic acid		
	Citric acid		
Inorganic base	Sodium hydroxide (NaOH)		^112^
Organic base	Guanidinium chloride (CH_6_ClN_3_)		^89^
Enzyme	Pepsin	Collagen extraction through cleavage of non-helical collagen extremities, breaking crosslinking	^13,30,36^
Physical treatment	Ultrasound	Improve the permeation and action of chemicals	^122^
	Microwave		^133^
	Heat		^112^
*Isolation and purification*			
Inorganic base	Sodium hydroxide (NaOH)	Isoelectric precipitation	^139,142^
Buffer solution	Tris (hydroxymethyl) aminomethane	Salting out precipitation and elimination of non-ColI constituents	^92^
Salt	Sodium chloride (NaCl)		^82^
	Ammonium sulfate (NH_4_SO_3_)		^143^
Physical treatment	Dialysis	Removal of salts	^127^
	Lyophilization	Drying	

Description of chemical species commonly applied in the pre-extraction, extraction, and isolation and purification steps for the obtention of Coll-I.

Although new technologies, such as deep eutectic solvent extraction, supercritical fluid extraction, or extrusion, have been reported^16,18^, the two most used methods to extract collagen consist of employing an acid solution or pepsin, (Tables 1 and 2). Different acids can be used for acidic extraction, such as lactic, citric, and acetic acid; the last is the most frequently used. Both acidic and enzymatic methods facilitate the dissolution of collagen and other tissue components. Although less used, Coll-I can also be extracted with basic solutions^19^. Physical treatments, such as heating, ultrasound, and microwave irradiation are employed to improve the effectiveness of chemical and enzymatic treatments, especially for extracting collagen from mammalian tissues. The selected extraction method is related to the Coll-I source. For instance, the acid method is most used for extracting Coll-I from aquatic sources (mostly fish), while pepsin is preferred for mammals (Table 1). After ColI-I extraction, a Coll-I-rich solution is obtained. Coll-I isolation and purification involve precipitating collagen by adjusting pH, while minimizing the precipitation of other proteins. This can be achieved by reaching Coll-I IP (discussed in detail further) in a process called isoelectric precipitation or adding salts in a process called salting out reaction, followed by salts removal through dialysis. The precipitated wet extColl-I is then filtered and lyophilized to obtain a dry sample, which is a stable form for storage for further uses. Solutions with different concentrations can be prepared from dried collagen.

### Relationships between amino acid (AA) composition of the extColl-I and the development of hydrogels

The content of AA in the extColl-I is directly related to the following factors, which will be addressed in detail further:

- AA contents can be used to evaluate the degree of enzymatic extraction and structural modifications induced by the extraction process, which will impact the gelation.
- The AA composition determines the IP of the extColl-l, which impacts solubilization and gelation.
- AA influences the denaturation temperature (T_d_) of the extColl-I, which is related to Coll-I thermal stability.
- The composition of lateral groups of AA is a key factor for crosslinking, blending, and preparing collagen-particle composites, which are used to tune extColl-I hydrogel properties.

### Effects of animal type and tissue in the chemical composition and structure of extColl-I

From this point, the main characteristics of the collagen macromolecules will be assumed as known. For readers less familiar with the structure of Coll-I^15,20^, please refer to the *Collagen type I: Basics* (Box 1) before proceeding.

ExtColl-I is usually composed of 19 AA, and the amount of each AA can differ depending on animal type and tissue, as shown in Fig. 2a, b, respectively. ExtColl-I from aquatic animal sources presented an average Gly percentage of 33 ± 1% while in mammals its average is 32 ± 1%, with no statistically significant difference among them (Fig. 2a). Considering the AA composition of native Coll-I, percentages close to 33% indicate a proper purity of extColl-I. The average imino acids (Pro and Hyp) content of extColl-I from aquatic animals (19 ± 2%) is significantly lower than for mammals (21 ± 4%), which is related to the lower Pro content (Fig. 2a). The hydroxylation of Pro (resulting in hydroxyproline, Hyp) provides thermal stability to collagen. The average amount of Hyp is comparable between extColl-I from mammals and aquatic sources. Significant variations in AA composition were found in different fish tissues, specifically for Gly, Pro, Asp, Ser, and imino acids. Lower Gly and Pro contents were found in extColl-I from scales compared to swim bladders (Fig. 2b). Hydroxylysine (Hyl) is an important hydroxylated AA that plays a crucial role in the structure of Coll-I and extColl-I. Hyl can form stable intermolecular crosslinking between collagen molecules and also serve as sites for carbohydrate attachment (glycosylation reaction). Collagen glycosylation might modulate cell adhesion and spread on basement membranes and is believed to be involved in the alignment of collagen fibrils in native tissues^21^. No significant differences in the average Lys and Hyl content were found comparing extColl-I from aquatic animals and mammals nor for the different fish tissues.

Box 1: Collagen type-I: Basics *Coll-I consists of three polymeric chains — two identical α1 chains and one α2 chain — each composed of repeating AA monomers. Each AA contains an amino (-NH*_*2*_*/-NH*_*3*_^*+*^*) and a carboxylic (-COOH/-COO*^*-*^*) group that react chemically forming amide groups, also know as peptide bonds* (Box Figure).

*The primary structure of Coll-I is characterized by the AA sequence Glycine(Gly)-X-Y. While X and Y are frequently proline and hydroxyproline, other amino acids, except tryptophan, can also occupy these positions*.

*The two α1 and one α2 chains form a triple helix as the secondary structure, with only the extremities of the chains — short segments known as telopeptides—remaining non-helical. Gly appears at every third position within the sequence, a pattern critical for forming Coll-I’s triple helix. The Gly-Pro-Hyp sequence is the most stabilizing tripeptide unit in the collagen structure*.

*One of the telopeptides extremity in the Coll-I chains has C-terminal groups, containing carboxylate/carboxylic acid groups and the other has N-terminal groups, containing deprotonated or protonated amino groups. These groups can bond chemically to other molecules, for examples when Coll-I is crosslinked and organized as fibrils*.

Box Fig. **Chemical-structural composition of Coll-I (Gly-X-Y)**. Central helical region of Coll-I and telopeptide regions containing C- and N-terminal extremities.

### Composition of extColl-I and extraction yield according to the extraction method

The AA composition of extColl-I can change depending on the extraction method. According to the literature, when collagen is extracted using pepsin, a reduction in Asp, Phe, and Tyr content is expected since pepsin presents cleavage sites in the telopeptides region, which are rich in these AA^22–24^. Extracted Coll-I missing the telopeptides region is called atelocollagen. Lower amounts of Asp, Phe, and Tyr were found for extColl-I with pepsin compared to acid extraction (Fig. 2c). Therefore, the quantification of these AA can be useful to follow the extent of the extraction process and the removal of telopeptides, which will influence extColl-I gelation (as discussed further). The use of a base solution, such as NaOH, in the pre-extraction or extraction of Coll-I leads to the conversion of asparagine and glutamine to aspartic (Asp) and glutamic acid (Glu), respectively^25^. Asparagine and glutamine are not identified or reported for extColl-I.

Coll-I extraction yield varies according to animal type, species, and tissue type (Fig. 3a). The pepsin extraction method yields higher extraction yields than acidic extraction (Fig. 3a). Typically, 0.5 M acetic acid and 0.05–1.5 wt % pepsin are used for Coll-I extraction^14,26–29^. However, higher acid concentrations, thus lower extraction pH, can lead to greater extraction yield due to more intense acid-catalyzed hydrolysis, as exemplified in Fig. 3b for Coll-I extracted from Asian bullfrog skin. Similarly, by increasing pepsin concentration, greater extraction yields are expected due to the increase of enzyme-substrate collision, substrate saturation, and diminished effect of possible inhibitory effect, as observed for the same Coll-I source in Fig. 3c.

**Table:** 

Summary: Coll-I extraction
• *In the last years, aquatic animals (mainly fishes) have been extensively studied for Coll-I sourcing for the design and development of hydrogels*.
• *Source of Coll-I affects the extraction method. Generally, Coll-I from mammalian sources requires harsher processes, like enzymatic extraction. On the other hand, non-mammalian sources can use gentler processes, such as acidic extraction, which better preserve the Coll-I structure*.
• *Variations in extColl-I AA composition were observed according to animal type and extraction method*.
• *Collagen type-I sourcing and extraction method strongly influence Coll-I extraction yield*.

## SECTION 2: ExtColl-I solubilization and gelation

The main steps in preparing hydrogels composed only of extColl-I are: (1) extColl-I solubilization in aqueous medium, (2) pH, and (3) temperature adjustment to propitiate gelation.

### Effect of aqueous solution pH on extColl-I solubilization and gelation

The pH is the most important parameter for extColl-I solubilization. As shown in Fig. 4a, at lower pH values, amine and carboxylic groups present in -N and C-terminal regions and from lateral groups from lysine (Lys)/histidine (Hys) and Asp/Glu AA are protonated (-NH_3_^+^ and -COOH), and the net charge is positive. At higher pH values, both amine and carboxylic groups are deprotonated (-NH_2_ and -COO^-^) and the net charge is negative. In these conditions, in which the residual charge is positive or negative, Coll-I molecules experience electrostatic repulsion and favorable solvation by water molecules and Coll-I can be partially or extensively dissolved in aqueous (polar) medium. ExtColl-I is more soluble at lower pH (acid medium) than at higher pH values (basic medium). That is why the protocols for Coll-I solubilization commonly use an acetic acid solution (at pH around 2), a weak acid used to disfavor acid-catalyzed collagen hydrolysis^30,31^. After solubilization of extColl-I in the desired concentration for the gel, the pH is increased to allow collagen gelation. At the pH at which the net charge is zero, i.e*.*, the IP, the solubility of collagen is the lowest since the electrostatic repulsion among molecules is minimized and Coll-I is more likely to aggregate.

As shown in Fig. 4b.1, different extColl-I samples can present different IP values, although most frequently IP is around 6–7. Such variations are related, among other factors (e.g*.*, sourcing), to acidic extraction methods or enzymatic treatments. For instance, acidic-extracted Coll-I presented lower IP values compared to pepsin-extracted Coll-I (Fig. 4b.2), and the Coll-I’s IP is shown to decrease with the enzymatic hydrolysis time, as exemplified for extColl-I from sheep skin digested with trypsin (Fig. 4b.3). The Coll-I’s IP, which is associated with the residual charge, is usually determined by zeta potential (ZP) analysis performed on Coll-I solutions at different pH values. The IP is the pH in which the ZP is zero. Similarly, variations in IP are observed depending on Coll-I sourcing. For instance, extColl-I from human amniotic membrane presented higher IP (*ca*. 8) than Coll-I from porcine skin (*ca*. 6) or starfish (*ca*. 5) (Fig. 4c.1); ExtColl-I from bones and scales presented higher IP (*ca*. 5) than muscles (*ca*. 4.6) and skin (ca. 4.4), for the same animal and species (Fig. 4c.2) and might differ from species to species (Fig. 4c.3).

Accordingly, extColl-I solubility varies according to the extraction method (Fig. 4d.1), animal tissue (Fig. 4d.1, d.2), and animal species (Fig. 4d.1, d.3). In the 4–6 pH range, pepsin-extColl-I presents greater solubility compared to acid-extracted Coll-I, which can be related to the shorter polymeric chains (Fig. 4d.1). Although the IP is the pH value at which collagen is more likely to form hydrogels, physiological pH (7.4) is often chosen when designing extColl-I hydrogels for applications in TERM.

### The effect of the ionic strength of the medium on extColl-I solubilization and gelation

**Fig. 5:**
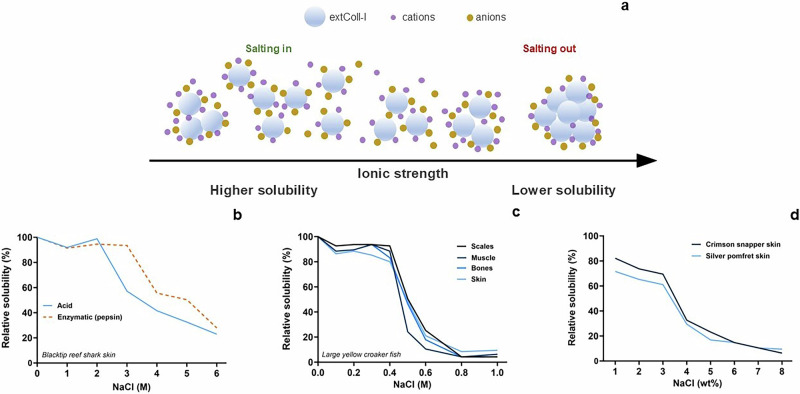
Effect of salts on Coll-I solubilization. Scheme demonstrating the solubility of extracted collagen type I (Coll-I) based on the ionic strength of the medium: Greater solubility occurs at lower salt concentrations due to the “salting in” effect, while lower solubility is observed at higher salt concentrations due to the “salting out” effect (**a**). Effect of ions on the solubility of extracted Coll-I from different extraction methods^121^ (**b**), animal tissues^37^ (**c**), and animal species ^92^ (**d**). **b**–**d** reproduce data shown in the cited articles in this caption.

In line with the process of mimicking physiological conditions, extColl-I hydrogels are generally prepared in electrolytic media, and extColl-I solubility is affected by the presence of electrolytes. This variation depends on the specific cation and anion of the electrolyte, the electrolyte concentration, and the characteristics of collagen, such as the content of chargeable groups and its molecular weight^32^. As presented in Fig. 5a, when salts are added in lower concentrations (e.g*.*, around 1–3 wt% of NaCl solution), a slight increase in solubility is observed due to the salting in effect^33^. For higher salt concentrations (e.g*.*, NaCl solution at 3 wt %^34,35^ or 0.4 mol/L^36,37^), the solubility of the extColl-I decreases proportionally with the increase in salt concentration due to the salting-out effect^33^. The solubility of the extColl-I also might vary depending on the extraction method, animal tissue, and animal species. For instance, the solubility of extColl-I from blacktip reef shark skin decreased in NaCl solutions with concentrations exceeding 2 M (Fig. 5b). In contrast, pepsin-extracted Coll-I maintained its solubility at NaCl concentrations up to 3 M, for the same animal and tissue (Fig. 5b). The concentration of NaCl affects extColl-I from different tissues differently. As an example, up to 0.4 M NaCl concentration, extColl-I from large yellow croaker fish skin presented lower solubility than the ones from scales, muscles, and bones. However, from 0.4 M, extColl-I from muscles experienced a more intense solubility decrease according to the NaCl concentration increase (Fig. 5c). ExtColl-I from Silver pomfret fish skin presented lower solubility than extColl-I from Crimson snapper fish skin. Both solubilities decreased with the increase in NaCl solution concentration from 1 wt% (Fig. 5c).

Papers studying variations in Coll-I solubility, according to salt concentration, usually employ NaCl salt even tough a more complex salt composition is usually found in cell culture media. As an example, standard Dulbecco’s Modified Eagle’s Medium presents a total concentration of inorganic salts lower than 0.2 M, whose respective concentration for NaCl did not considerably affect Coll-I solubility. If a more concentrated medium is used for cell-laden hydrogels, salts’ concentration effect should be taken into consideration since Coll-I solubility state could play a role in hydrogels’ physical stability and performance.

### The effect of temperature on extColl-I solubilization and gelation

Temperature is a key factor that promotes collagen solubilization^38^ and gelation^39^. However, solubilization and the adjustment in pH of the pre-gel are frequently performed at low temperatures (e.g*.*, in an ice bath) to prevent Coll-I denaturation and premature gelation. If the denaturation temperature (T_d_) of extColl-I is reached or surpassed during heating, the tertiary structure of the protein is partially or completely lost, which can affect hydrogel structuration. As discussed by Sarrigiannidis et al.^12^, finding trends on gelation temperature and mechanical properties is not straightforward and results from literature pointing at opposite trends are found. For instance, Tang et al.^40^ studied the effect of heating treatment on pepsin-extracted Coll-I from chicken feet and its effect on hydrogel strength. Heating Coll-I at 70 °C increased strength compared to 50 °C, suggesting that moderate heating can promote gel network formation. However, higher temperatures weakened the gel attributed to extensive collagen denaturation and thermal degradation.

**Fig. 6:**
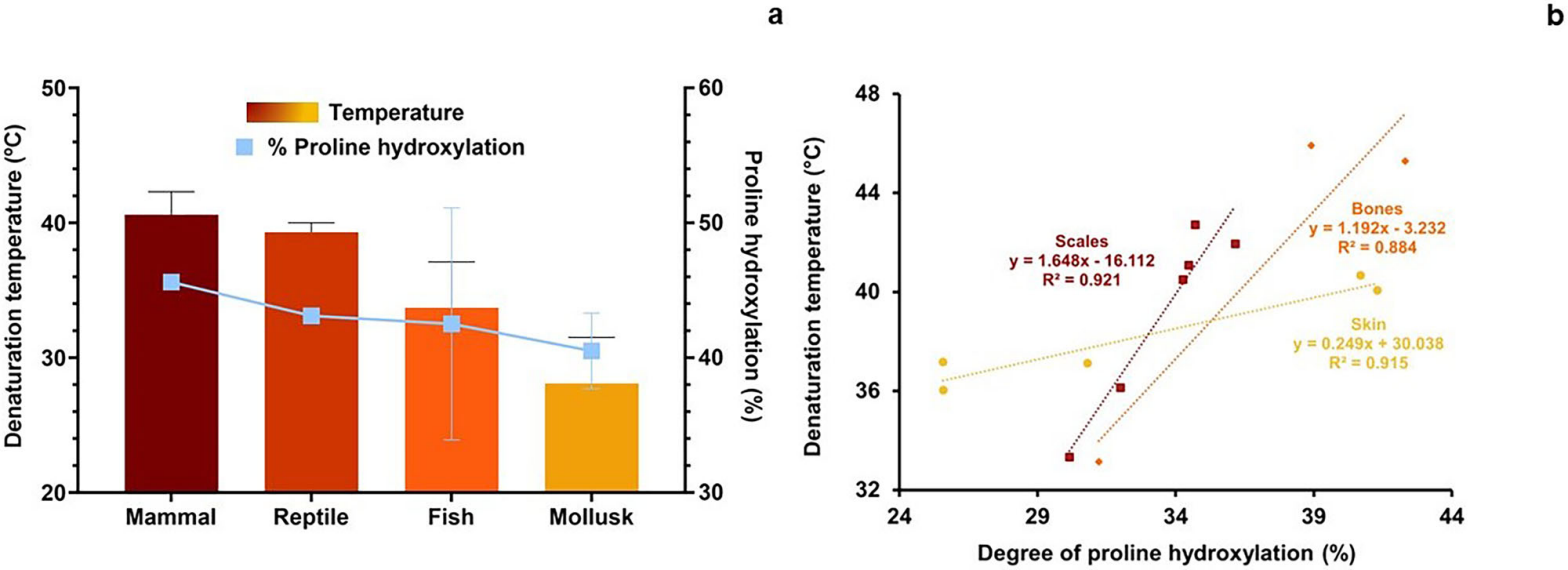
Relation between proline (Pro) hydroxylation and denaturation temperature (T_d_) for Collagen type I (Coll-I) extracted from different animals and tissues. Differences in T_d_ for Coll-I extracted from different animal types^26,37,95,100,104,109,117,124,126,127,129^(**a**); relation between T_d_ and the percentage of Pro hydroxylation depending on the animal tissue^37,89,100,109,117,124,127^ (**b**). * *p*: < 0.1, **: *p* < 0.05, and ***: *p* < 0.01. Data was collected from references cited in this caption.

The T_d_ of the extColl-I is related to the degree of hydroxylation of Pro to Hyp, which occurred in vivo during tissue formation. The greater the degree of Pro hydroxylation, the greater the extColl-I T_d_ (Fig. 6a). T_d_ varies with animal (T_d_ mammal > T_d_ reptile > T_d_ fish > T_d_ mollusk) and tissue type, as shown in Figs. 6a, b, respectively. The differences in T_d_ among different animals (especially fish) can be related to the temperature of their habitats. For instance, extColl-I from cold-water fishes have lower T_d_ than the extColl-I from hot-water fishes^41–43^.

### Parameters influencing gelation kinetics of extColl-I hydrogels

**Fig. 7:**
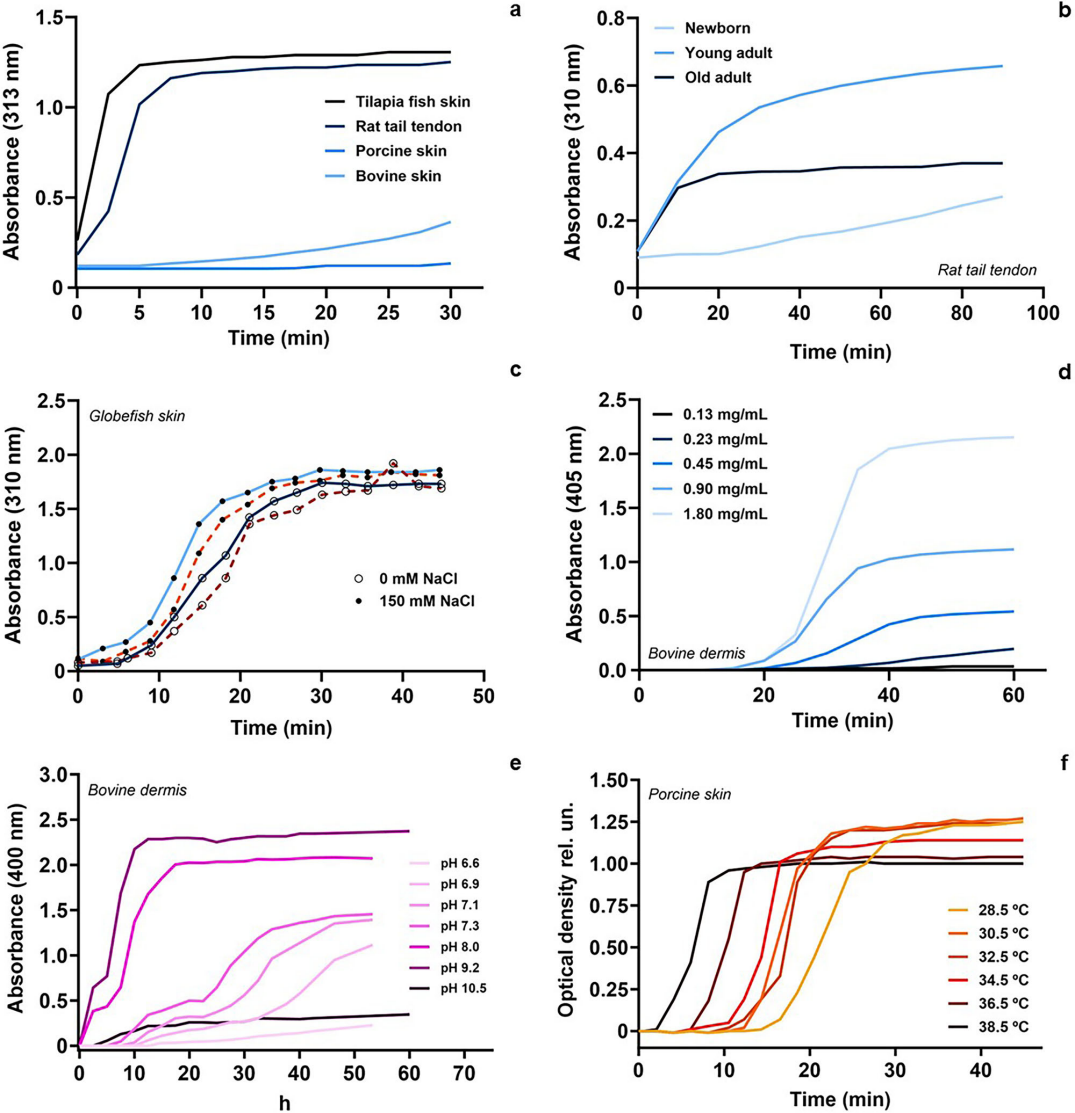
Effect of different variables on the gelation kinetics of Coll-I. Gelation kinetics of Coll-I depending on animal type^162^ (**a**), animal age^163^ (**b**), extraction method (acid in blue and pepsin in orange) and ionic strength of the medium^164^(**c**), Coll-I concentration^165^(**d**), pH^44^(**e**), and the temperature of the medium^39^(**f**). **a**–**f** reproduce data shown in the cited articles in this caption.

The parameters discussed so far, such as animal tissue, pH, and ionic strength of the medium, among others, affect extColl-I gelation kinetics. Some examples are illustrated in Fig. 7. Both animal type and age affect gelation, as shown in Figs. 7a, b, respectively. ExtColl-I from both young and old animals tends to exhibit an inferior capacity for fibers reassembling. Respect to the extraction methodology, acid-extracted Coll-I might undergo more rapid gelation compared to pepsin-extracted Coll-I (Fig. 7c), which could be related to the preservation of telopeptides. In the same figure, it is shown how gelation is also affected by the ionic strength of the medium, as it correlates with the solubility of the extColl-I^44^: faster gelation was observed in a medium with 150 mM NaCl compared to the absence of additional salts.

The reassembly of extColl-I fibers is favored at higher polymer concentrations (Fig. 7d), and it is privileged at pH values close to the IP of extColl-I^45^, since it is promoted by hydrophobic interactions, as exemplified in Fig. 7e^44,46^. Below and above the IP, repulsion between positive and negative charges, respectively, hinders fibers reassembling^45^.

Interestingly, Coll-I can form hydrogels at around 37 °C, which is the temperature of the human body. Thus, the extColl-I pre-gel can be injected and only become a hydrogel after application. Small variations around the physiological temperature might affect gelation (Fig. 7f).

### Parameters influencing mechanical and biological properties of extColl-I hydrogels

Assessing the mechanical and biological properties of Coll-I hydrogels is essential for validating and optimizing them for application in specific tissues. Like most biological tissues, hydrogels exhibit both viscous and elastic behavior when undergoing deformation^47^. They can be physically understood as a biphasic system composed of a solid-like polymeric networking surrounded by an aqueous solution^47^. Consequently, when stress is applied, their physical response consists of two components: A resistance to shear flow (viscous component) and a linear strain with time under stress (elastic component).

Several mechanical properties of hydrogels can be evaluated. For instance, the elastic modulus gives information on hydrogels’ capacity to deform elastically under stress (e.g*.*, compressive, tensile, shear, bending, or twisting; In the case of compressive or tensile stress along one direction, the elastic modulus is called “Young modulus”). The storage and complex modulus, referred to as G’ and G*, respectively, are related to the energy stored by the hydrogel under oscillatory deformation^48^. The greater the modulus, the stiffer the hydrogel.

Like hydrogels, cells can also respond to time-dependent forces applied in their environment. That is why mechanical and biological properties are related^48^. The biological performance of hydrogels can be assessed through in vitro^49^ and in vivo^50^ tests, performed outside or inside a living organism, respectively. Most of Coll-I hydrogel’s biological characterization in literature consists of in vitro tests. In vitro essays can involve various processes and functions that enable the cell to perform its specific role in the organism when in the presence of the hydrogel^49^. For instance, cell morphology, adhesion, migration, proliferation, viability, and metabolism can be evaluated through cells, products of cell metabolism, and nutrient quantification or microscopy analysis. However, the understanding of the effects of the Coll-I source (i.e*.*, animal type and tissue), the extraction method used, and the physical-chemical characteristics of extColl-I on the mechanical properties and biological response of extColl-I hydrogels remains limited. One reason for this limitation could come from the high complexity involved in such evaluation when numerous parameters affect the performance of hydrogels. This could explain the low number of articles relating the composition and structure of the extColl-I used in hydrogel preparation with their mechanical and biological properties.

Our systematic search for Coll-I hydrogels from January 2018 to December 2023, revealed that most articles still focus on characterizing them in terms of primary structure and physical-chemical parameters. Therefore, data presented from this point about the mechanical and biological performance of Coll-I hydrogels are provided as examples, and the generalization of them is discouraged.

**Fig. 8:**
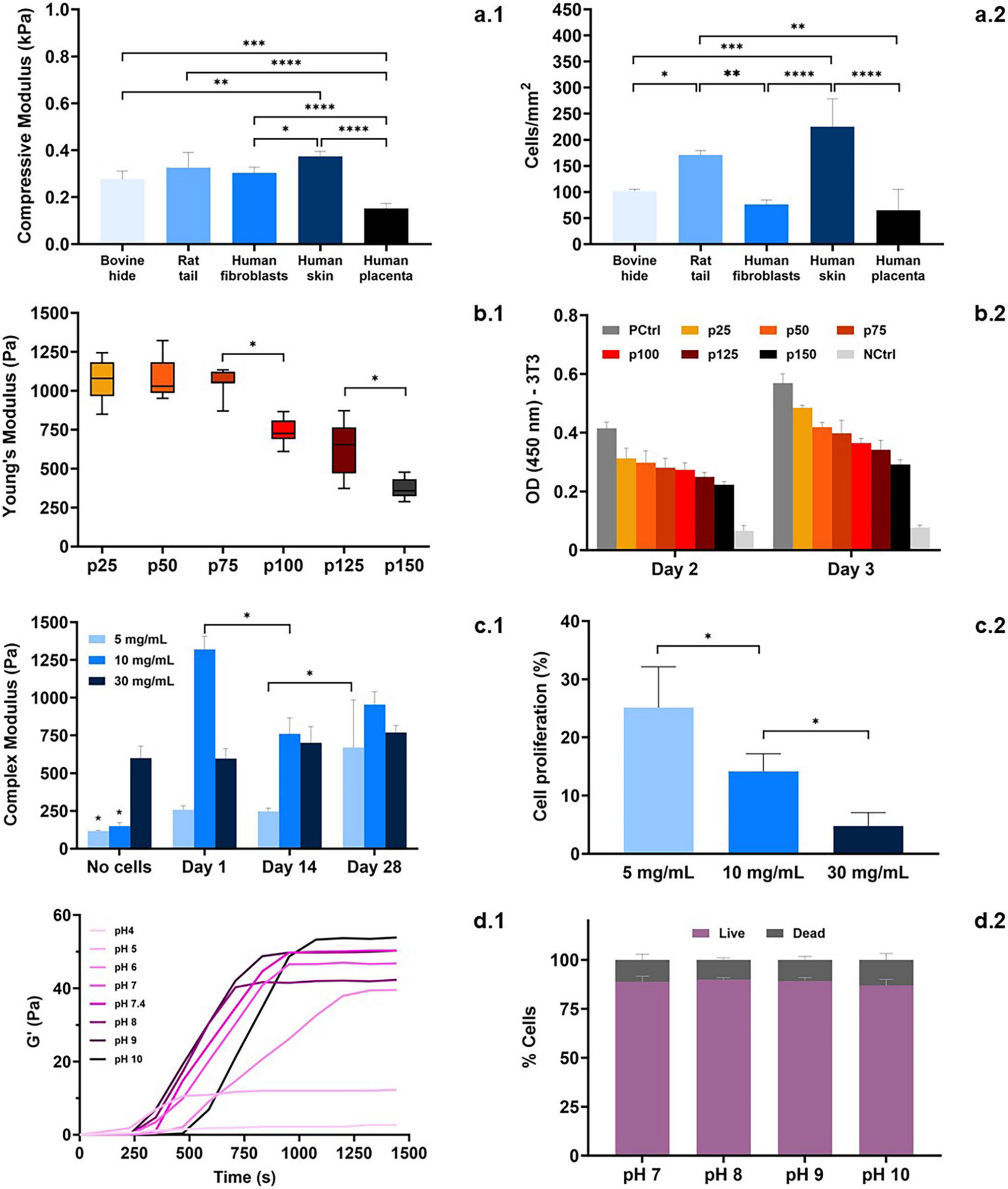
Effect of different variables on mechanical and biological properties of Coll-I hydrogels. Examples of how parameters, such as animal type and tissue^166^(**a**), extraction method^30^(**b**), collagen concentration^167^(**c**), and pH of the medium^168^(**d**) can influence mechanical properties (1) and biological behavior (2) of collagen type I hydrogels. OD: optical density.* *p*: < 0.05, **: *p* < 0.01, and ***: *p* < 0.001. **a**–**d** reproduce data shown in the cited articles in this caption.

As shown in Fig. 8a.1, a.2, the source of Coll-I (animal type and tissue) might result in hydrogels with different stiffness (by measuring the compressive modulus) and different cell proliferation properties, respectively. Regarding the extraction method, telopeptides, the non-helical extremities of Coll-I that can be removed during collagen extraction using pepsin, could serve as important structural elements to obtain more resistant hydrogels (Fig. 8b.1), and provide a more favorable environment for cell adhesion, spread, and proliferation (Fig. 8b.2).

The relation between Coll-I concentration in cell-laden hydrogels with mechanical performance could not be linear. Cells can contract the hydrogel exerting tensile forces^51^ and deposit ECM, potentially increasing its stiffness. However, cells can also secrete enzymes that remodel the collagen matrix in early stages (Fig. 8c.1). Additionally, stiffer and more resistant hydrogels, with denser polymeric structures, may hinder cell proliferation^52,53^ and cell-mediated hydrogel remodeling, thereby reducing the impact of cells on mechanical properties (Fig. 8c.2). Improvements in both mechanical properties and biological performance could encounter certain limitations if these variables respond to Coll-I concentration in different directions.

As previously mentioned, the pH of gelation will influence the net charge of the extColl-I, which is related to its solubility and gelation kinetics. As shown in Fig. 8d.1, the stiffness of the hydrogel can also be influenced by the pH. In this example, stiffness was improved when the pH was close to or above the IP of the extColl-I. In cell-laden hydrogels prepared at physiological or slightly basic pH, which have undergone external densification, cells appear to survive regardless of the pH used for gelation (Fig. 8d.2).

**Table:** 

Summary: Solubilization and gelation of the extracted Coll-I
• *Isoelectric point and denaturation temperature are parameters related to the structure and AA composition of the extracted Coll-I which ultimately is related to the extraction method employed and the chosen animal type and tissue*.
• *The extracted collagen type-I is more likely to dissolve in solutions with pH values below or above the isoelectric point (especially in acidic medium). On the other hand, Coll-I precipitation and gelation are privileged at pH values close to the IP*
• *By increasing the ionic strength of the medium, the extracted collagen might experience a slight increase in solubility due to the salting-in effect (for low salts concentration) or an intense decrease in solubility due to the salting-out effect (for high salts concentration)*.
• *Gelation kinetics is influenced by several parameters, such as Coll-I sourcing, extColl-I AA composition and concentration, pH and ionic strength of the medium, and temperature*.

## SECTION 3: Enhancing the structural properties of extColl-I hydrogels

ExtColl-I hydrogel’s stiffness can be improved and tuned through three main techniques: (1) Crosslinking the polymer chains, (2) blending extColl-I with other polymers, and (3) adding nano- or microparticles to form composites.

Coll-I crosslinking consists of connecting groups present in Coll-I’s AA laterals chains and at the extremities of the Coll-I chains or adding molecules that strengthen the interactions between the collagen protein chains and fibers (associations of Coll-I triple helices) by establishing intermolecular forces (e.g., hydrogen bonds, dipole-dipole interactions) or by forming chemical bonds (covalent). On the other hand, Coll-I blending combines Coll-I solution with another continuous polymeric phase to originate a homogeneous solution. In the third approach, hydrogels stiffness is modified by the addition of nano- or microparticles to the pre-gel generating a suspension in which particles are entrapped in the hydrogel after gelation. The presence of bioactive particles can provide additional properties to the hydrogels. These systems, in which the extColl-I is a continuous phase containing an additional solid phase, are called composites in this work.

### Common crosslinkers and chemical reactions between lateral groups on extColl-I and crosslinkers

**Fig. 9:**
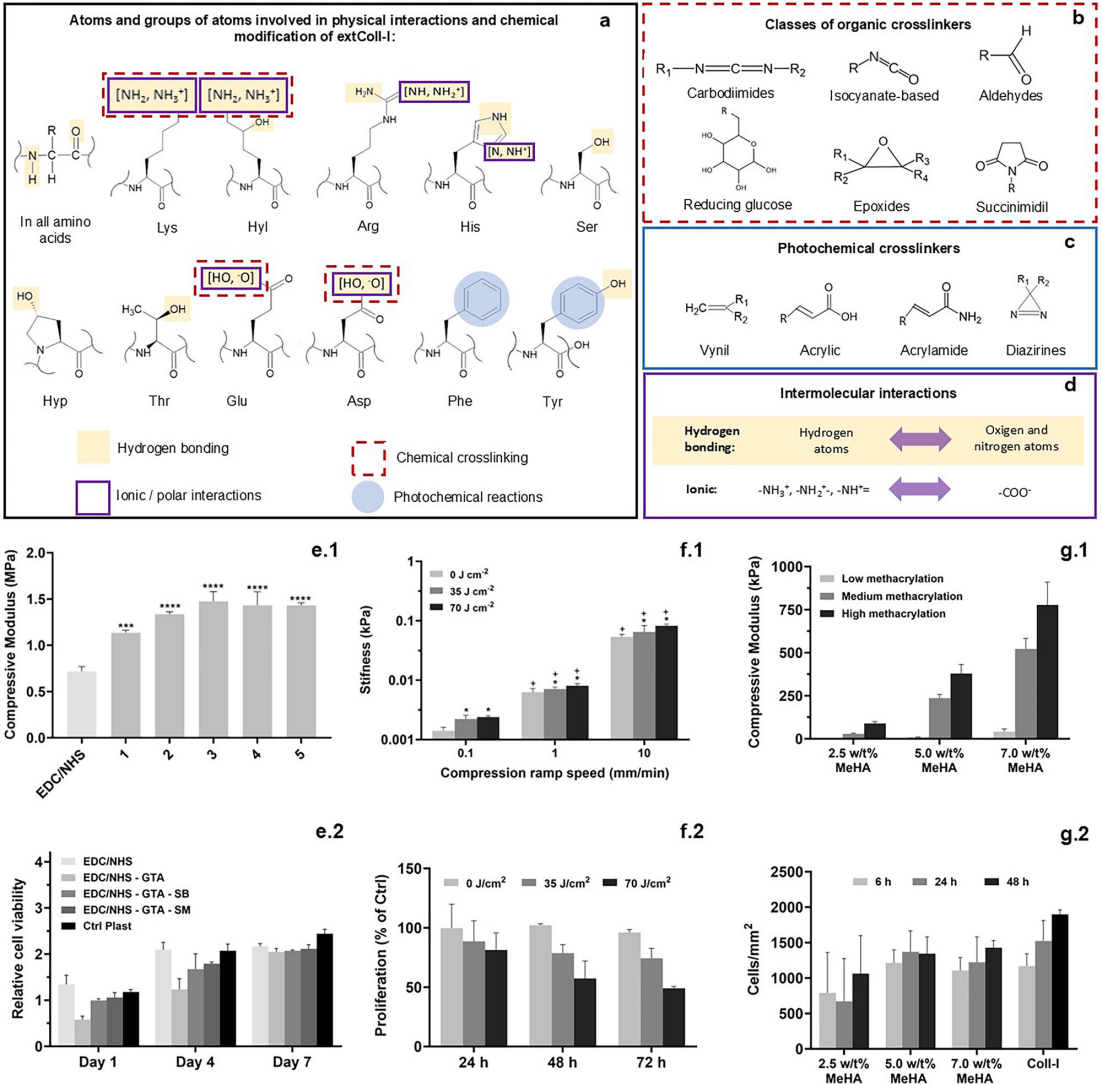
Crosslinked hydrogels. Lateral groups in amino acids of Coll-I able to establish hydrogen bonding, ionic interactions, and chemical and photochemical crosslink reactions (**a**). Classes of organic (**b**) and photochemical (**c**) crosslinkers. Details of hydrogen bonding and ionic interactions on Coll-I (**d**). Examples of the effect of chemical (**e**)^169^ and photochemical (**f**^64^ and **g**^170^) crosslinking in mechanical properties (1) and biological performance (2) of Coll-I hydrogels. In (**e**) Hydrogels were crosslinked with 1-ethyl-3-(3-dimethylaminopropyl) carbodiimide hydrochloride (EDC):N-hydroxysuccinimide (NHS) and double crosslinked with EDC:NHS and different glutaraldehyde:Coll-I molar ratios (0.018:1, 0.18:1, 0.45:1, 0.9:1, and 2.7: are samples 1, 2, 3, 4, and 5, respectively). Not-crosslinked residues of glutaraldehyde were neutralized with sodium borohydride (SB) or sodium metabisulfite (SM). Ctrl Plast: Control in culture plastic. * and + *p*: < 0.05, **: *p* < 0.01, and ***: *p* < 0.001. In (**f**) * and + indicate significant differences between UV-C and compression ramp speed conditions groups, respectively. **e**–**f** reproduce data shown in the cited articles in this caption.

In collagen, chemical groups in the C-terminal and N-terminal, as well as some AA within the chains containing carboxylic and amine-based lateral groups, are reactive to chemical crosslinkers that form strong covalent bonds between extColl-I chains (Fig. 9a).

Among the different types, organic crosslinkers can be classified according to their organic functional groups as carbodiimides, epoxides, aldehydes, succinimides, isocyanate-based compounds, and reducing glucose agents (Fig. 9b). The general reactivity of each of these classes of crosslinkers will be discussed in detail below.

Carbodiimides react with acid groups^54,55^, while aldehydes (e.g*.*, glutaraldehyde^56^) and epoxies (e.g*.*, 1,4-Butanediol diglycidyl ether^57^) react with N-containing groups, such as Lys and Hyl. In the case of carbodiimides, the reaction with carboxyl groups, as in the AA residues of Asp and Glu, relies on succinimidyl species (such as disuccinimidyl glutarate) to form reactive ester groups by activation of Lys and Hyl lateral groups. Isocyanate-based crosslinkers (such as hexamethylenediisocyanate) are reactive with chemical groups containing active hydrogen atoms, such as hydroxyl (-OH), carboxylic acid (-COOH), and N-containing groups (-NH_2_, -NH-) present in collagen. However, it will react preferably with primary amines, such as the ones present in Lys and Hyl^45^. Finally, reducing glucose, such as glucose-6-phosphate^58^, participates in glycation reactions with amine-containing AA, such as Lys and Hyl. Other crosslinkers with unique chemical structures and function can be cited, such as genipin^59^ and the enzyme lysyl-oxidize^60^.

Photochemical crosslinkers^61,62^ are molecules used to obtain photoreactive extColl-I and can generally be classified as vinyl, diazirines, acrylamide, or acrylic-containing organic molecules (Fig. 9c). When extColl-I reacts with these species, double bonds reactive to UV light are added to the protein allowing the radical formation of covalent bonds. However, collagen can be photochemically crosslinked in the absence of a crosslinker. ExtColl-I by itself has aromatic side chains in Phe and Tyr that can originate radicals when treated with UV radiation (Fig. 9a). The combination of these radicals in the collagen structure leads to intermolecular crosslink formation^63–65^.

ExtColl-I hydrogels can also be structured by adding chemical species that can interact through hydrogen bonds with N and O atoms present in the polymeric chain, as well as lateral groups of AA containing -OH *(*e.g*.*, Hly, Hyp, Ser, Thr, and Tyr), carboxylic (Glu and Asp), imino (His), guanidino (Arg), and amine groups (Lys and Hly), as highlighted by the yellow squares in (Fig. 9a).

When the lateral group of the AA Lys, Hyl, Arg, His, Glu, and Aps are charged, they can establish ionic interactions through physical crosslinks (Fig. 9d). A residual charge on Coll-I occurs mainly at pH values below the dissociation constant (pKa)^66^ values of lateral groups of Lys, Hly, Arg, and His (equal to 10.53, 9.5^67^, 12.49, and 6.00, respectively), and above the pKa values of Glu and Asp (equal to 4.25 and 3.65, respectively).

### The effect of crosslinking on mechanical and biological properties of extColl-I hydrogels

Crosslinking strategies are employed to improve the stiffness of collagen hydrogels. However, the hydrogel’s performance can be affected. Some examples of the effect of crosslinking Coll-I-based hydrogels on mechanical properties and biological performances are presented below.

As exemplified in Fig. 9e.1, the addition of a chemical crosslinker like glutaraldehyde (GT) improved the compressive modulus of previously crosslinker 1-ethyl-3-(3-dimethylaminopropyl) carbodiimide hydrochloride:N-hydroxysuccinimide Coll-I hydrogels. In this work, although GT improved the compressive modulus of Coll-I hydrogels, the cytotoxicity of the crosslinker was demonstrated. Alternatively, GT inhibition using sodium metabisulfite and sodium borohydride led to reduced cytotoxicity (Fig. 9e.2).

For photochemical crosslinking of collagen without crosslinker molecules, UV power and exposure time are crucial parameters, as they dictate the density of the crosslinks formed. As shown in Fig. 9f, the average stiffness of Col-I hydrogels increased when irradiated with UV-C, with a further increase observed as the UV-C radiation energy was raised from 35 to 70 J/cm² (Fig. 9f.1). However, fibroblast cell proliferation decreased with UV-C irradiation, and this effect intensified with higher energy levels (Fig. 9f.2).

Fig. 9g shows an example of UV crosslinked Coll-I with methacrylate hyaluronic acid. The compressive modulus of the resulting gels increased as the methacrylate level of hyaluronic acid also increased (Fig. 9g.1). The high stiffness of the higher methacrylate hyaluronic acid concentrations and the cell adherent properties of the collagen resulted in greater cell adhesion and proliferation, comparable to the hydrogel of collagen (Fig. 9g.2).

Amirrah et al.^15^, Kong et al.^68^, and Copes et al.^69^ references are indicated for additional and complementary information to the discussion performed above about crosslinking strategies for Coll-I.

### Polymers and particles for blended and composite extColl-I hydrogels

ExtColl-I can be blended with polymers soluble in aqueous medium (usually natural polymers). These polymers include gelatin (hydrolyzed collagen), gellan gum, silk fibroin, hyaluronic acid, chitosan, agarose, alginate, and cellulose-base polymers, as shown in Table 3.

**Table 3:** Coll-I blended hydrogels

Coll-I concentration	Additional polymer(s)	Additional polymer(s) concentration	Blending method	Remarks	Ref.
5 (w/v)%	Type A gelatin and poly (ethylene glycol) diacrylate (PEGDA)	10 (w/v)% (gelatine) and 5–60 (v/v)% (PEGDA)	Gelatin and collagen were added to PBS solution (pH 7.4) with the subsequent addition of PEGDA.	Formulations were tested as inks. Lower concentrations of PEGDA presented better print accuracy print fidelity, and printability.	^144^
1 mg/mL	Gellan gum (GG)	0.4 (w/v)%	Gellan gum and MgCl_2_ (Mg^2+^ cations were used as physical crosslinkers) aqueous solutions were separately prepared (at 90 °C). These solutions were then mixed to neutral Coll-I solution.	Steam cells laden hydrogels were prepared. The hydrogel enabled steam cells’ spreading and proliferation and promoted fibroblast migration.	^145^
0.5–4 mg/mL	Silk fibroin (SF)	1:2.5, 1:3.5, 1:5, 1:7, 1:10 Coll-I:SF weight ratio	Coll-I neutral solution was mixed with previously ultrasonicated SF aqueous solution.	The progressive increase in Coll-I concentration in the SF hydrogels showed Coll-I provides adhesive sites to steam cells, stimulating cell binding and proliferation.	^146^
2 mg/mL or lower	Hyaluronic acid (HA)	3.75 mg/mL (initial)	Coll-I solution, solution of HA reacted with EDC/NHS crosslinker, and oxidized bacterial cellulose golden nanoparticles suspension were mixed.	Bacterial cellulose/gold nanoparticles enhanced hydrogel elastic modulus and decreased the gelation temperature. The hydrogel presented a self-sealing response due to reversible physical crosslinking.	^147^
15 mg/mL or lower	Chitosan (CH)	100/0, 75/25, 50/50/, 25/75, and 0/100 Coll-I/CH volumetric ratios.	Coll-I and CH acidic solutions were prepared separately and then mixed.	Coll-I and CH showed synergism to improve thermomechanical properties and cell viability of the hydrogels.	^148^
2 mg/mL	Agarose (AGA)	2 and 4 (w/v)%	Neutral Coll-I solution was mixed with previously dissolved AGA solution (dissolved in PBS and autoclaved at 120 °C).	Chondrocyte cells were added to the hydrogel. Coll-I allowed cell proliferation and continual glycosaminoglycan production.	^149^
25 mg/mL or lower (initial)	Dialdehyde carboxymethyl cellulose (DCMC)	1:2, 1:1.6, 1:1.2, 1:0.8, and 1:0.4 DCMC: Coll-I weight ratios	Coll-I acidic solution was mixed with DCMC solution (with crosslinking capacity).	Greater crosslinking density was obtained for Coll-I richer hydrogels. Coll-I improved mechanical properties, thermal denaturation temperature, and degradation resistance of the hydrogels. Fibroblasts were compatible with all hydrogels compositions.	^150^
2.5 mg/mL	Alginate	10 mg/mL	Alginate was dissolved in water and mixed with Coll-I solution. Hydrogels were treated in CaCl_2_ solution, which provides Ca^2+^ cations (ionic crosslinker) to alginate.	Neuron cells adhered to the blended hydrogel, formed a branched neural network, expressing a protein involved in neurotransmission. Mechanical properties of the hydrogel were tunned by modulation of the Ca^2+^ ionic crosslinker concentration. Alginate ionic crosslinker concentration also influenced neuron-specific gene expression.	^151^

Compilation of papers detailing Coll-I and the additional polymer(s) concentrations, a resume of the blending method employed, and remarks.

**Fig. 10:**
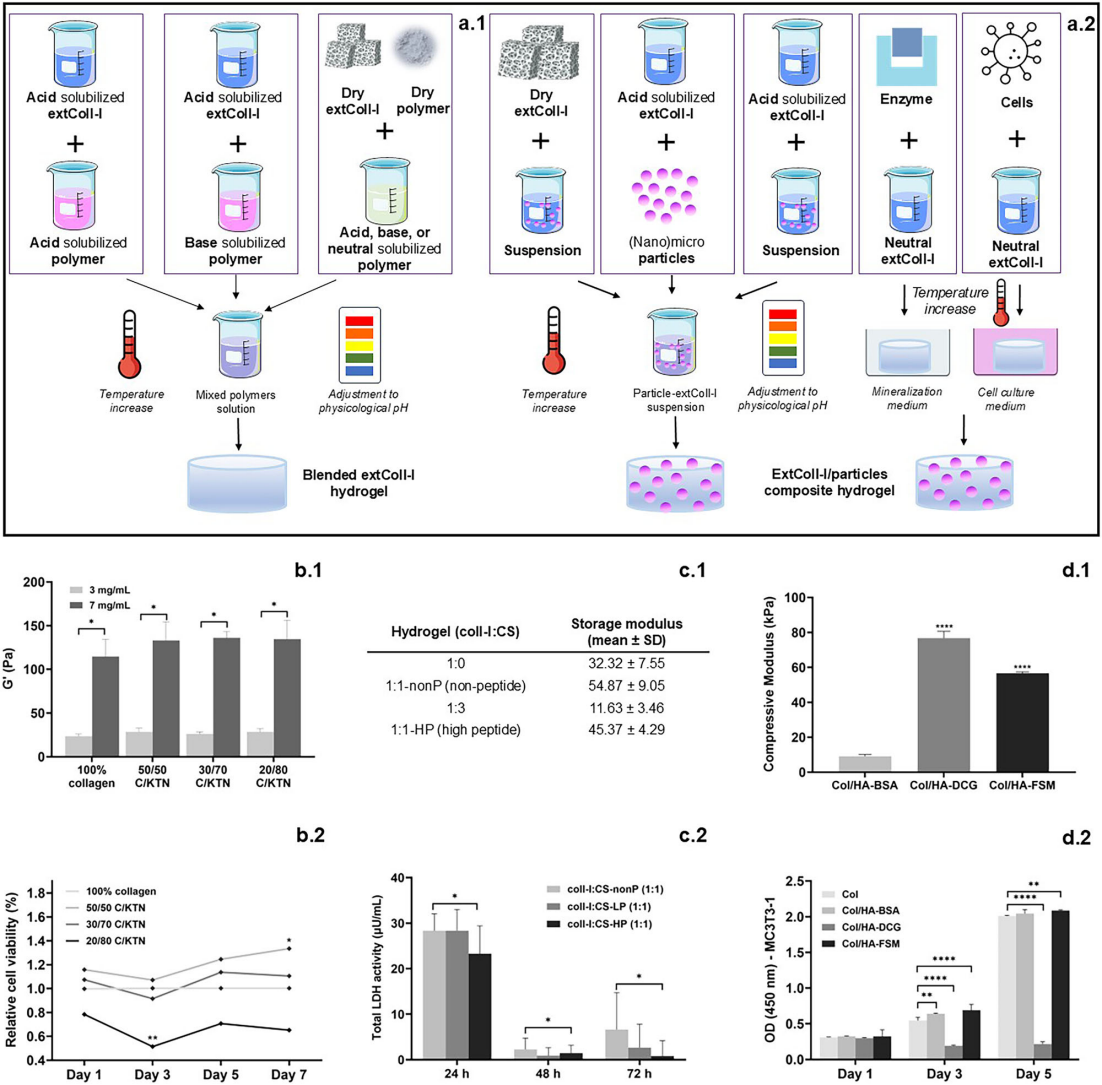
Blended and composite hydrogels. Steps for developing blended-collagen type I hydrogels and collagen type I-particles composite hydrogels (**a**). Examples of mechanical (1) and biological (2) properties of collagen type I blends (**b**^76^ and **c**^77^) and collagen type I composite hydrogels^78^ (**d**). C/KTN collagen/keratine, CS-LP and -HP chitosan-low peptide and -high peptide, LDH lactate dehydrogenase, HA-BSA hydroxyapatite – blending self- assembling method, DCG diffusion and crosslinking with glutaraldehyde method, FSM freezing, salting-out, mineralization method. **b**–**d** reproduce data shown in the cited articles in this legend. *:*p* < 0.05, **: *p* < 0.01, and ***: *p* < 0.001. **a** was partly generated using Servier Medical Art, licensed under Creative Commons Attribution 4.0.

General blending steps include (Fig. 10a.1): (1) Solubilizing extColl-I in acid, the additional polymer in acid, neutral, or basic solution, and (2) mixing both polymers, and (3) adjusting the pH close to the physiological one (*c.a*. 7.4) and increasing the temperature. Note that the two polymer solutions can also be at neutral pH before the mixture and that, although less practiced, both dried Coll-I and the additional polymer can be solubilized directly in the same solution. In Table 3, a compilation of papers on Coll-I blended hydrogels is presented, detailing the Coll-I and the additional polymer(s) concentrations, a resume of the blending method employed, and remarks that include the improvement of Coll-I hydrogels’ mechanical and/or biological properties when they are blended.

Although few papers report the level of integration between Coll-I and the additional polymer, the formation of a physical interconnected network was reported by Buitrago et al.^70^, for Coll-I/Silk fibroin, and Branco da Cunha et al.^71^ for Coll-I/alginate (crosslinked with Ca^2+^) hydrogel blends. Additionally, Wilharm et al.^72^ investigated directional interactions between Coll-I and elastin fibers, which was suggested to consist of a parallel alignment; the presence of elastin led to the formation of larger and similarly oriented domains compared to pure Coll-I hydrogels.

Particles, such as metallic (micro)nanoparticles, oxides, layered solids, carbonaceous materials, and polymeric particles (usually synthetic polymers presenting low solubility in water and, thus, unable to form blends with extColl-I) can be added to the extColl-I pre-gel composition to form composite hydrogels.

Four general methods were identified for particles incorporation into Coll-I-based hydrogels, as shown in (Fig. 10a.2). (1) Dry extColl-I can be solubilized into particle suspension, (2) dry particles can be added to Coll-I solution, (3) both Coll-I solution and particle suspension can be mixed, or (4) particles can be generated in situ (still less practiced in literature). Particles homogeneous dispersion is an important condition for acquiring homogeneous composite hydrogel. In this sense, dispersive physical methods (such as ultrasound treatment) can be employed.

Table 4 provides examples of Coll-I composite hydrogels, a resume of the respective methods for encapsulating particles, and presents remarks on mechanical and biological properties provided by particles to the hydrogels. As described in Table 4, apatite particles can be enzymatically mineralized into Coll-I hydrogels^73^ and hydroxyapatite particles can be biomineralized using cell activity^74^. In a more recent approach, metallic silver (Ag) nanoparticles were generated in situ from Ag^+^ cations photoreduction^75^.

**Table 4:** Composite Coll-I hydrogels

Coll-I concentration	Particles nature	Particles concentration	Processing technique for particles incorporation into Coll-I hydrogels	Remarks	Ref.
3 mg/mL	Apatite	16.7 or 22.6 (particles∕polymer weight ratio)	Apatite was mineralized in situ under hydrogel incubation in calcium-glycerophosphate in the absence or presence of the enzyme alkaline phosphatase at 1.25 or 2.5 mg/mL.	Mineralization led to stiffened hydrogels.	^73^
3 mg/mL	Hydroxyapatite (Hap)	N/A	Hap mineralization was induced by human adipose stem cells in bioactive glass (experimental glass 2-06) osteogenic medium.	Mineralized Coll-I promoted the osteogenic differentiation of stem cells encapsulated in the hydrogel and osteocalcin production.	^74^
1–5 wt %	Hydroxyapatite nanopowder (HapNP)	0–40 w/v %	HapNP was added in Coll-I pre-gel solution, containing genipin crosslinker.	Coll-I hydrogel containing hydroxyapatite was compatible with stem cells cultivated inside.	^152^
2.5, 4, and 6 mg/mL	Laponite nanoclay (LapNC)	0.1 w/v % LapNC culture medium was used to dilute Coll-I.	LapNC was dispersed in the cell culture medium, which was used to dilute Coll-I pre-gel solution.	LapNC improved the stiffness of Coll-I microenvironment, which was shown to affect tumor cell growth and aggregation.	^153^
7 mg/mL	Bacterial nanocellulose fibers (BCf)	74 wt % Col-I and 26 wt % BCf.	BCf suspension was mixed with Coll-I pre-gel solution.	BCf improved the stiffness of Coll-I hydrogel while maintaining its viscoelasticity. Coll-I hydrogel supported steam and fibroblast cells inside.	^154^
1.6 wt %	Polyvinylpyrrolidone (PVP) capped zinc oxide nanoparticles (ZPVP)	1: 0.25, 1: 0.5, and 1: 1 (Coll-I: ZPVP) weight ratios	ZPVP suspension was mixed with Coll-I pre-gel solution.	PVP was used as a physical crosslinker between Coll-I and ZPVP. Particles improved hardness, adhesiveness, injectability, and rheological properties of the hydrogels.	^155^
4 mg/mL	Silver (Ag) nanoparticles	0.1 and 0.5 mg/mL	AgNO_3_ salt (containing Ag^+^ cations) was added to Coll-I pre-gel solution. Ag nanoparticles were generated in situ by exposing Ag^+^ to visible light, which propitiated Coll-I photochemical crosslinking.	The hydrogel presented improved injectability, enhanced biological activity, and broad-spectrum antimicrobial properties.	^75^
NA	Silver (Ag) nanoparticles	0, 100, 200, 300, 400, 500, and 600 ppm	Lyophilized collagen was reconstituted in Ag nanoparticles aqueous solution.	Ag nanoparticles–Coll-I hydrogel accelerated wound healing.	^156^
3 mg/mL	Carboxylate fluorescent polystyrene microbeads (CFPM)	∼1.455 × 10^6^ particles per hydrogel	Silica or beads were mixed with 10X PBS and the suspension was added to Coll-I pre-gel solution.	Coll-I fibrils bound to the CFPM, which acted as nucleation sites.	^157^
2.4 mg/mL	Carboxylic single-walled carbon nanotubes (CNT)	0, 0.5, 1, and 2 wt %	CNT were added to Coll-I pre-gel solution, which was sonicated to improve particles dispersion.	CNTs enhanced cardiomyocyte adhesion and elongation.	^158^

Compilation of papers on collagen type I composite hydrogels, detailing collagen and particle concentrations, a resume of the methods employed for particles in situ particles generation or particle incorporation in the hydrogels, and remarks.

### Effects of polymer blending and (nano)particle incorporation in extColl-I Hydrogels

In the literature, several works can be found on polymer blending and particle incorporation in Coll-I hydrogels to enhance their mechanical and biological performance and adapt them to the desired application. In some cases, a polymer, or particles, is not only added to improve mechanical properties of the Coll-I hydrogel but also to modify other parameters such as stability and to enhance bioactivity.

For instance, it is observed in Fig. 10b.1 that Coll-I concentration can improve hydrogels’ mechanical properties. Furthermore, at the biological level, the use of keratin appeared to prevent tumoral cell proliferation in a cell-laden hydrogel, likely due to the increased matrix density (Fig. 10b.2)^76^. In Fig. 10c, chitosan modified with a peptide binding motif specific for cardiomyocytes was blended with collagen observing an increase in the storage modulus on the collagen : chitosan hydrogels (Fig. 10c.1). Furthermore, the presence of the peptide motif together with collagen decreased the cell death in a cell-laden hydrogel after 48 h of culture (Fig. 10.c2), likely due to enhanced cell attachment^77^.

Finally, a case of a collagen composite that also combines crosslinking is illustrated in Fig. 10d^78^ on a mineralized Coll-I/hydroxyapatite hydrogel. Three methods for synthesizing the hydrogel were tested: (1) Preparing a suspension of salts and Coll-I, allowing collagen to self-assemble; (2) First forming a chemically crosslinked Coll-I hydrogel and then allowing salts to diffuse in; and (3) using an innovative approach involving freezing, salting out, and mineralization, which allows for salts diffusion into a frozen Coll-I hydrogel. Each method resulted in hydrogels with different mechanical properties, with strength being highest in the hydrogels created from frozen Coll-I solutions and those that were crosslinked (Fig. 10d.1). However, the enhanced strength of the crosslinked hydrogel may have been more due to the crosslinking effect rather than mineralization. In terms of cell viability, the first and third methods yielded the best results, while the use of glutaraldehyde negatively impacted cell viability due to the toxicity of this molecule (Fig. 10d.2).

**Table:** 

Summary: Enhancing the structural properties of Coll-I hydrogels
• *ExtColl-I’s structure can be modified by crosslinking through reactive amine and carboxylate groups from AA, leading to modifications in mechanical properties and biological performance*.
• *ExtColl-I can be combined with additional, usually natural, water-soluble polymers forming blended systems to tune mechanical and biological properties*.
• *Similarly, (nano)particles (e.g., metallic, layered, 3D solids) can be combined to extColl-I, forming composite systems to tune Coll-I hydrogels properties*.

## Conclusions and future perspectives

In this work, the entire process of obtaining a collagen hydrogel was revisited, from the selection of the animal source to tuning Coll-I hydrogels’ properties. Several interrelations permeating the different steps of this process were identified. Consequently, key parameters such as collagen source, extraction method, amino acid composition, isoelectric point, ionic strength of the medium, and temperature emerged as critical factors. These parameters were shown to affect Coll-I processing and hydrogel performance, highlighting their interdependence.

Only a few studies in the literature relate Coll-I (and collagen types in general) extraction and gelation with the mechanical and biological performance of hydrogels. Indeed, up to now, the research on Coll-I comprises mainly one of the aspects: Compositional and structural characterization or mechanical and biological validation. Complete papers evaluating Coll-I from extraction to hydrogel development and validation are needed. In this way, variations from sample to sample and methodologic limitations can be overcome, allowing assessing relationships between Coll-I *sources-extraction method-chemical composition-structure-mechanical properties-biological performance*. Studies like that and the one presented in this work are expected to contribute to the fine-tuning of Coll-I hydrogel properties for TERM applications.

## Data Availability

All data generated or analyzed during this study are included in this published article: References used for reproduced graphs and data extracted, treated, and shown in tables and as graphs in the current study are cited in the legend of the respective figures.

## List of abbreviations

AA: Amino acids
Ala: Alanine
Arg: Arginine
Asn: Asparagine
Asp: Aspartic acid
Cys: Cysteine
Coll-I: Collagen type I
T_d_: Denaturation temperature
ExtColl-I: Extracted collagen type I
Gln: Glutamine
Glu: Glutamic acid
Gly: Glycine
His: Histidine
Hly: Hydroxylysine
Hyp: Hydroxyproline
IP: Isoelectric point
Ile: Isoleucine
Leu: Leucine
Lys: Lysine
Met: Methionine
Phe: Phenylalanine
Pro: Proline
Ser: Serine
Thr: Threonine
Trp: Tryptophan
Tyr: Tyrosine
Val: Valine
